# Influence of Elevated Temperature on Color Centers in LiF Crystals and Their Photoluminescence

**DOI:** 10.3390/ma16041489

**Published:** 2023-02-10

**Authors:** Małgorzata Sankowska, Pawel Bilski, Barbara Marczewska, Yaroslav Zhydachevskyy

**Affiliations:** 1 Institute of Nuclear Physics, Polish Academy of Sciences PAN (IFJ PAN), Radzikowskiego 152, 31-342 Kraków, Poland; 2 Institute of Physics, Polish Academy of Sciences, 02-668 Warsaw, Poland

**Keywords:** lithium fluoride, FNTD, photoluminescence, color centers, temperature

## Abstract

The radiation-induced photoluminescence (PL) of LiF has found its way into many applications for the detection and imaging of ionizing radiation. In this work, the influence of thermal treatment at temperatures up to 400 °C on absorption and PL emission spectra as well as fluorescent nuclear tracks in irradiated LiF crystals was investigated. It was found that carrying out PL measurements with the crystals kept at the temperature of about 80 °C leads to a considerable increase in luminescence emission of F_3_^+^ color centers at 525 nm. This enhancement of PL intensity allows for the microscopic imaging of the fluorescent nuclear tracks using only F_3_^+^ emission, which is not possible at room temperature. It was also found that heating the irradiated crystals before measurement at temperatures from 100 °C to 200 °C increases the concentration of F_3_^+^ centers. However, the related enhancement of PL emission is insufficient in terms of enabling the observation of the fluorescent tracks in this part of the spectrum. In the case of the main PL emission at 670 nm related to F_2_ centers, the thermal treatment at around 290 °C substantially increases the intensity of fluorescent tracks. This effect, however, was found to occur only at low fluences of alpha particles (up to about 10^9^ cm^−2^); therefore, it is barely visible in the emission spectrum and not noticeable in the absorption spectrum.

## 1. Introduction

Lithium fluoride (LiF) is a well-known optical and luminescent material. One of its most interesting features is its generation of color centers during exposure to ionizing radiation and photoluminescence (PL) related to these centers. In LiF, ionizing radiation creates mostly F centers (anion vacancy with one electron), which can later aggregate into more complex defects such as F_2_ (two anion vacancies connected with two electrons) and F_3_^+^ (three anion vacancies connected with two electrons) color centers. These two types of color centers, which, in the absorption spectrum, show a common band at c.a. 445 nm (called M band), are especially interesting as they emit strong luminescence while excited with blue light. The emission spectrum consists of two main bands: green with a maximum of near 525 nm corresponding to F_3_^+^, and red with a maximum of around 670 nm corresponding to F_2_ [1].

Despite the fact that color centers in LiF and their photoluminescence have been studied for decades, these subjects still attract a great deal of scientific interest, wherein the objective is the acquirement of a better understanding of various effects and the development of new applications [2,3,4,5,6,7]. Radiation-induced photoluminescence offers wide applicational opportunities for the detection, dosimetry, and imaging of ionizing radiation [8,9,10,11,12,13]. One of the most recent achievements in these areas is the visualization of the tracks of single particles by using a fluorescence microscope in the technique called Fluorescent Nuclear Track Detection (FNTD) [14]. The FNTD method demonstrated its usefulness for such purposes as alpha particle spectroscopy [15], neutron detection [16], ion beam measurement [17], cosmic radiation measurement [18], and others. Although this technique seems to have great potential, it suffers from a low signal-to-noise ratio. At the moment, even for the brightest tracks, the background noise is about 20% of the maximum track intensity. Therefore, the enhancement of the signal-to-noise ratio and PL intensity is very important, as this could enable imaging tracks that are currently not visible and open new directions of applications. It seems that a feasible way in which to increase the signal is by using heat treatment. Some research indicates that post-irradiation annealing at temperatures of 200–250 °C causes an increase in the intensity of the photoluminescence signal [19]. Others reported an increase in concentrations of complex color centers after thermal annealing at 130 °C [20,21]. Such effects might be explained by the enhancement of F_2_ and F_3_^+^ concentrations caused by the interaction of single F centers and smaller aggregates with anion vacancies released from complex color centers [20,22].

Although the existence of the influence of temperature treatment on the absorption and photoluminescence emission of lithium fluoride crystals has been known for a long time, the works published so far on this subject still leave some questions unanswered. One such question is the influence of the temperature on the PL signal of crystals irradiated with very low doses. Moreover, many of the reported results seem to contradict each other. For example, some researchers claim that F_2_ centers disappear at lower temperatures than F centers [22,23], while others have observed entirely opposite behavior [20,21]. These discrepancies might be caused by differences in heating and cooling profiles, heat treatment duration, and the individual characteristics of the LiF crystals used. In addition, irradiation conditions play an important role, as it has been proven that the concentration of F_2_ and F_3_^+^ centers created in LiF depend strongly on the temperature at which the crystal has been irradiated. The investigations are usually performed on LiF crystals irradiated with very high fluences of ions [20,21] and neutrons [24] or very high doses of gamma radiation [25]. As we demonstrated in our previous studies [26], the radiation dose has a significant influence on the presence of thermal effects. Thermal behavior may also be heavily affected by the type of ionizing radiation used.

In our previous work, we described the enhancement of the intensity of the fluorescent tracks caused by a heat treatment at temperatures up to about 300 °C applied to lithium fluoride crystals following their irradiation [26]. While we did observe such an increase in the signal for very small doses (single particles), the opposite effect, i.e., a decrease in the fluorescent signal, was observed for high doses or high particle fluences. These results prompted us to conduct a more in-depth analysis of the absorption and emission spectra obtained after subjecting irradiated LiF crystal samples to heat treatment. The research was performed for two different kinds of radiation, alpha and beta, and two different types of heat treatment. We also compared the spectral results with microscopic images of the tracks observed for samples irradiated with small fluences of alpha particles.

## 2. Materials and Methods

**Figure 1:**
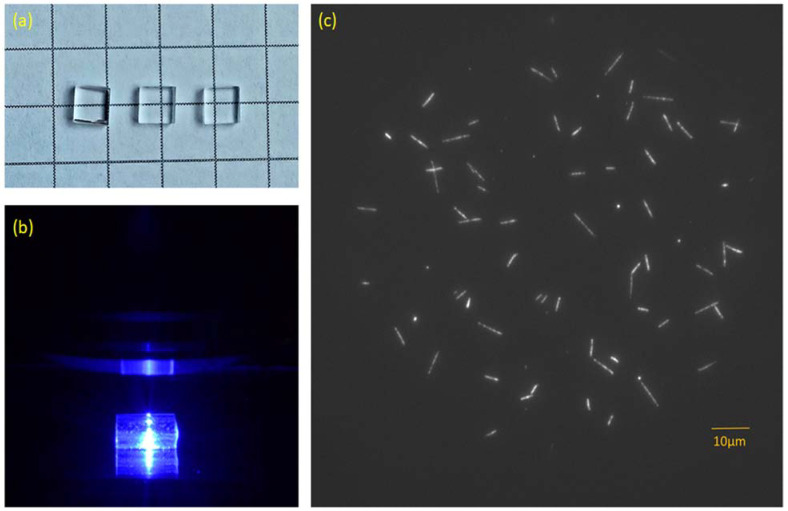
(**a**) LiF crystal samples after being cut and polished; (**b**) magnified image of a LiF sample illuminated with blue light (440 nm) during read-out with Nikon Eclipse wide-field microscope; (**c**) examples of fluorescent tracks registered at RT after irradiation with alpha particles from Am-241 source (particle fluence around 10^6^ cm^−2^). The images present maximum intensity projection from 15 images acquired with focus set at different depths of the crystal in 1 μm steps.

LiF single crystals were grown with the Czochralski method at the Institute of Nuclear Physics in Kraków. For all samples used in our studies, pure, undoped LiF powder was used as a starting material. The grown crystals were later cut using diamond saws into small samples of a standard size of 4 × 4 × 1 mm (Figure 1a). Samples were polished with abrasive straps and rinsed in acetone in an ultrasonic washer. Before its first use, each sample was annealed for ten minutes at a temperature close to the melting point of LiF (around 820–830 °C) in order to improve the quality of the sample’s surface. This procedure removes small scratches that are created during polishing.

Irradiation of the crystal samples was performed with alpha or beta radiation. As an alpha source, the Am-241 AMRB5718 source with activity of 10.7 MBq produced by Eckert&Ziegler (Berlin, Germany) was used. The nominal energy of Am-241 alpha-particles is 5.486 MeV, but due to the composition of the source (thick layer of the active material and 2 µm thick covering gold foil), the energy was degraded to about 3 MeV [15]. The irradiations of the samples intended for spectra measurements were carried out without any collimators, while irradiations of samples for which microscopic images were taken were carried out using a special metal collimator to ensure a nearly perpendicular direction of the particles with respect to the sample’s surface. The used collimator had a thickness of 6 mm and a round hole with a diameter of 2 mm. Beta irradiations were conducted using a Sr-90/Y-90 source with a dose rate of 0.324 kGy/h. After irradiation, we waited at least 24 h before performing any measurements as it has been previously demonstrated that the photoluminescence signal may change within the first few hours after irradiation [27,28].

Spectral measurements were carried out on the irradiated samples, and these underwent two different kinds of heat treatment, which will henceforth be referred to as Method 1 and Method 2:

- Method 1—A LiF sample after irradiation was submitted to step-annealing, i.e., a series of subsequent heat treatments at increasing temperatures from room temperature (RT) up to 400 °C with a step of 20 °C. After each heating step, the sample was cooled to RT and emission or absorption spectra were registered. Then, the sample was heated again to the next temperature step and the whole procedure was repeated.
- Method 2—PL emission spectra were measured while raising the temperature at a linear ramp. The heating stage was mounted into the setup of the fluorescence microscope. The sample was placed at the stage at RT. Then, the heat was turned on, and the sample was heated to 400 °C with a heating rate of 50 °C/min. At the same time that the heating procedure was employed, the sample was illuminated with 440 nm light and emission spectra were registered every 2 s.

These two methods provide information on slightly different kinds of processes that affect color centers. Method 1 concerns a stationary situation; therefore, it detects only long-lasting effects. Method 2, on the other hand, deals with the dynamic situation, thereby enabling the observation of instantaneous processes as well.

Photoluminescence emission spectra were measured with the QE65 PRO (Ocean Optics, Orlando, FL, USA) spectrometer mounted on a Nikon Eclipse Ni-U fluorescence wide-field microscope instead of the CCD camera. As the excitation light source, a pE-100 illumination system with 440 nm LEDs (CoolLED, UK) was used together with band-pass filter ET445/30 (Chroma Technology, Bellows Falls, VT, USA). Measurements were performed using a 5× TU Plan Epi objective lens and a long-pass 515lp filter. Heating of the samples during emission spectra measurements was performed using the THMS600 heating stage (Linkam Scientific, Redhill, UK). For step-annealing (Method 1), each treatment was started by placing the sample at the heating stage at room temperature. Then, it was heated to the set temperature with a heating rate of 150 °C/min and the set temperature was maintained for 3 min. This heat treatment time was found to be optimal in our previous studies. After this time elapsed, the heating was turned off and the sample was cooled slowly, remaining at the heating stage for another 3 min.

Absorption spectra were measured with Varian Cary 5000 UV-Vis-NIR spectrophotometer (Agilent Technologies, Santa Clara, CA, USA) at the Institute of Physics, Polish Academy of Sciences in Warsaw. The measurements were only conducted for Method 1, as performing the measurements at elevated temperatures (Method 2) was not possible due to technical reasons. The heating of the samples was realized using a TL reader. The heating rate was 300 °C/min and each sample was heated for 3 min at a set temperature before the heating was turned off.

Besides spectra measurements, microscopic observations of the fluorescent tracks of ionizing particles were also carried out using Nikon Eclipse Ni-U wide-field fluorescence microscope (Nikon, Tokyo, Japan) together with CCD DS-Qi2 camera (Nikon, Tokyo, Japan) (Figure 1b,c). Images were taken using a 100× TU Plan ELWD (NA 0.80) objective lens and different emission filters: a long-pass filter ET570lp (Chroma) and a bandpass filter HQ 535/30. The field of view was limited by a diaphragm and had a quasi-circular shape with a diameter of about 90 μm. All microscopic images were taken at a depth of 3 µm beneath a sample’s surface. Computer analysis of the obtained images was performed using ImageJ software (with Fiji interface) Version 1.53t [29].

Track intensity was calculated as the maximum intensity in a track after background subtraction. For calculations, we only considered tracks with almost circular shape as they originate from particles that are nearly perpendicular to the sample’s surface. Thus, we were able to eliminate the influence of particles’ angle of incidence on track intensity. The background signal was calculated as a modal value of intensity in the circle of a radius of 50 pixels (c.a. 3.5 μm) around a track.

## 3. Results and Discussion

### 3.1. Method 1

#### 3.1.1. Absorption Spectra

**Figure 2:**
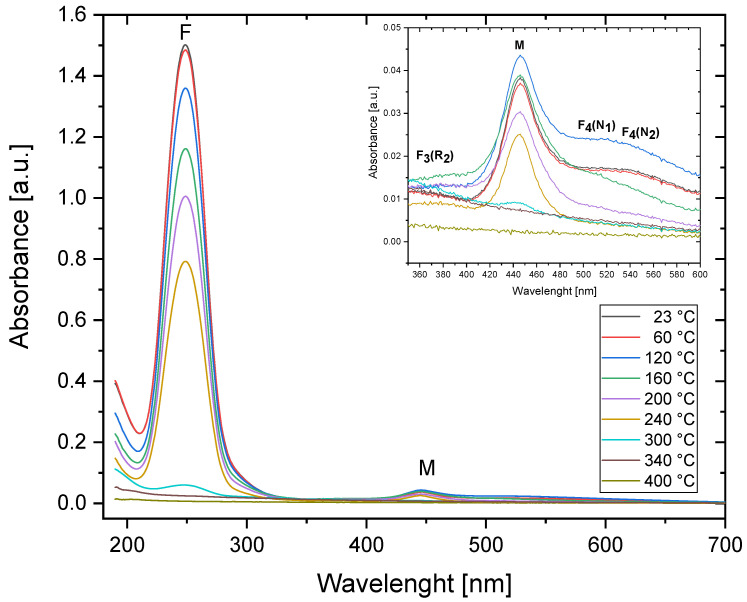
Absorption spectra for the sample irradiated with 30 kGy of beta radiation (Sr-90/Y-90) and submitted to a series of subsequent heat treatments at increasing temperatures (Method 1). Measurements were taken at room temperature.

**Figure 3:**
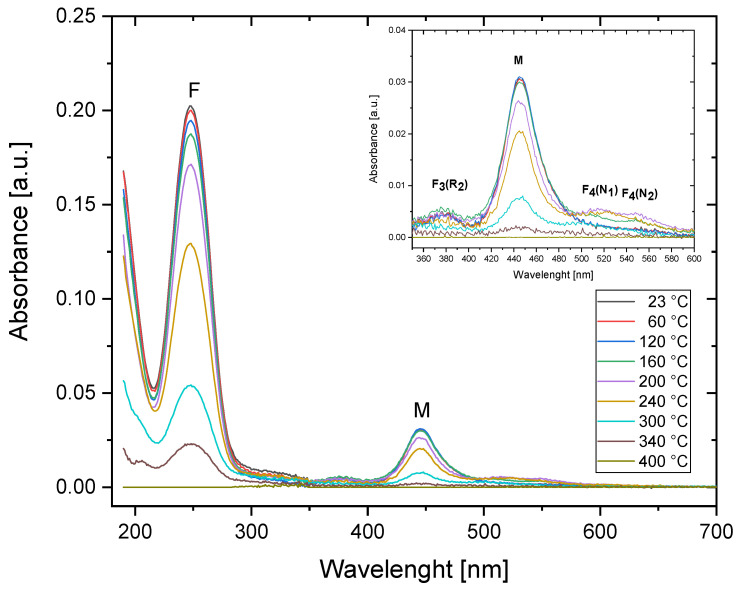
Absorption spectra for the sample irradiated with Am-241 alpha particles (fluence 3.4 × 10^12^ cm^−2^) and submitted to a series of subsequent heat treatments at increasing temperatures (Method 1). Measurements were obtained at room temperature.

The series of absorption spectra measurements for Method 1 was conducted both for the sample irradiated with 30 kGy of Sr-90/Y-90 beta radiation and for the sample irradiated with Am-241 alpha particles with 3.4 × 10^12^ cm^−2^ fluence. The measured absorption spectra are presented in Figure 2 and Figure 3. The strongest bands are marked on the graphs with the names of the color centers that they originate from. For both types of radiation, the most prominent feature is the peak at a wavelength of around 250 nm, which is related to F centers. The M band, consisting of two overlapping bands related to the F_2_ and F_3_^+^ color centers, is visible around 445 nm. Peaks associated with other, more complex color centers are barely visible in the absorption spectra; therefore, they will not be discussed further.

Comparing the spectra registered at RT with respect to the beta and alpha irradiations, one can see that while the overall absorbance is higher for the beta-irradiated sample, the ratio of M/F bands is higher in the case of the alpha-irradiated crystal. This means that the ratio of the total concentration of F_2_ and F_3_^+^ color centers to the concentration of F centers is higher for the sample irradiated with alpha radiation. This may be explained by analyzing the manner in which the dose is deposited in the case of both radiation modalities. Beta particles penetrate a small LiF crystal in an almost uniform way. Oppositely, the range of 3 MeV alpha particles in LiF is only 9.3 µm. As alpha particles deposit their energy only in the sub-surface part of the sample, the energy transferred by them per unit volume of the crystal is much higher. Converting the alpha particle fluence of 3.4 × 10^12^ cm^−2^ to the average dose absorbed within a 9.3 µm thick layer of LiF yields the value of 224 kGy, which is much more than the 30 kGy used for beta irradiations. Moreover, each single alpha particle deposits energy in a highly non-uniform way. In the core of a track, which has a nanometer size, the dose is extremely high, reaching the megagray range and decreasing very steeply in accordance with the distance from the core. The probability of the formation of complex defects increases with the growing concentration of the primary F centers; therefore, higher doses increase the number of F_2_/F_3_^+^ color centers with respect to the number of F centers [30].

**Figure 4:**
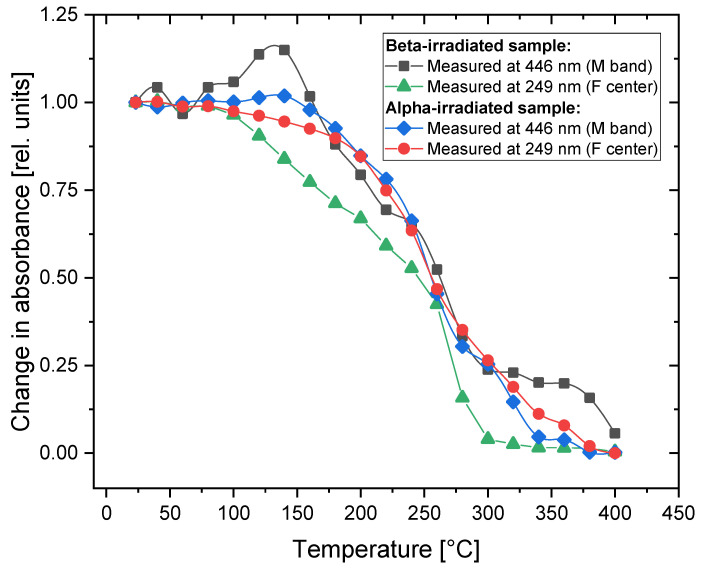
Relative changes in the absorbance in the two most prominent peaks of the spectrum (at 446 nm and 249 nm) measured after applying the treatment according to Method 1 for the beta-irradiated (30 kGy) and alpha-irradiated (fluence 3.4 × 10^12^ cm^−2^) samples.

Relative changes in the absorbance measured for the wavelengths at which the maximum intensity of the M band (at 446 nm) and the peaks corresponding to the F centers (249 nm) were registered and are presented in Figure 4. Regarding the alpha-irradiated samples, no significant increase in absorbance was observed after applying Method 1. The absorbance levels measured for both F and M bands remains the same up to 150 °C but decrease in a similar manner above that temperature for both bands. The changes observed in the absorption spectra measured for the crystal irradiated with beta radiation are slightly different from those measured for the alpha-irradiated sample. There is a small increase in absorbance measured for the M band (at 446 nm) after heating at temperatures from 100 °C to 160 °C with the maximum at 140 °C. This temperature of the heat treatment agrees quite well with the annealing temperature after which the increase in the complex color centers’ concentration noticed by Dauletbekova et al. occurs [20]. In addition, the peak related to the F centers disappears faster for the beta-irradiated samples, and at a temperature of 300 °C, it can no longer be measured.

#### 3.1.2. PL Emission Spectra

**Figure 5:**
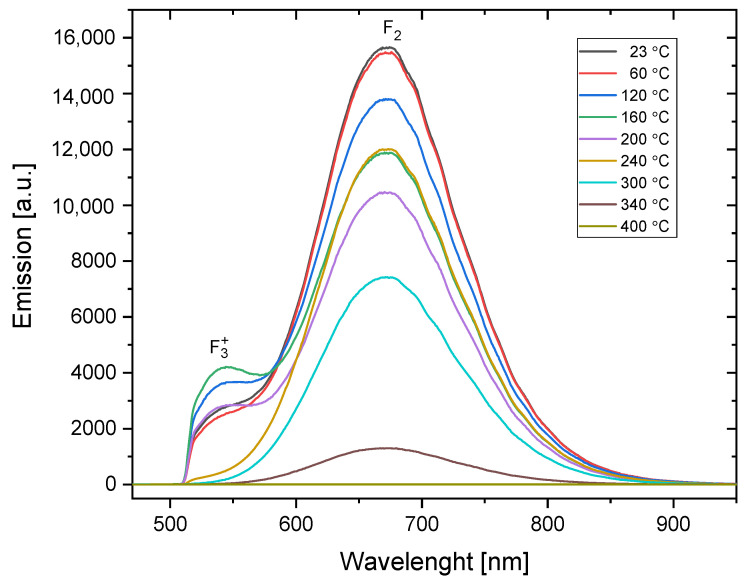
PL emission spectra for the sample irradiated with 30 kGy of beta radiation (Sr-90/Y-90) and submitted to a series of subsequent heat treatments at increasing temperatures (Method 1). Excitation was applied at 440 nm. The bands related to F_2_ and F_3_^+^ color centers are marked. Measurements were obtained at room temperature. The spectrum is clipped from the low-wavelength side by the applied filter.

**Figure 6:**
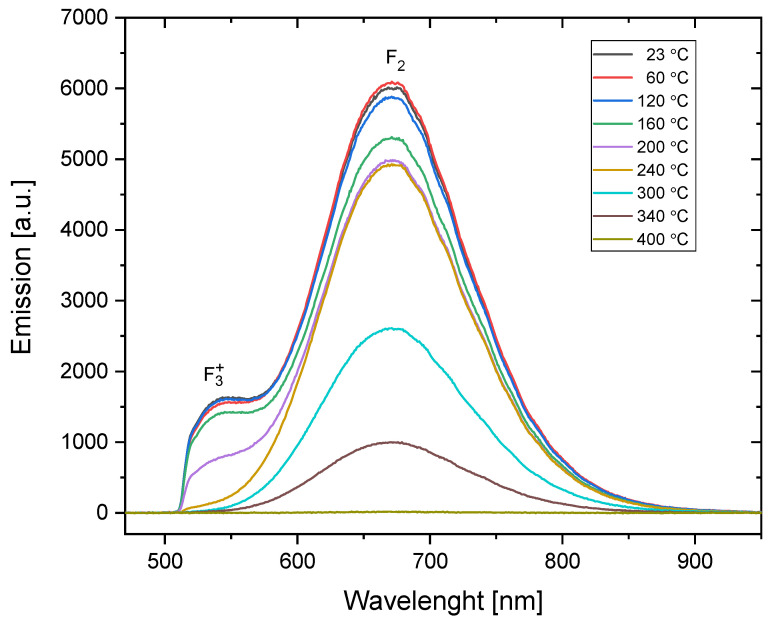
PL emission spectra for the sample irradiated with alpha particles from Am-241 source (fluence 3.4 × 10^12^ cm^−2^) and submitted to a series of subsequent heat treatments at increasing temperatures (Method 1). Excitation was applied at 440 nm. The bands related to F_2_ and F_3_^+^ color centers are marked. Measurements were obtained at room temperature. The spectrum is clipped from the low-wavelength side by the applied filter.

The photoluminescence emission spectra measured for the samples irradiated with the dose and particle fluence identical to those used for the absorption spectra measurements are presented in Figure 5 and Figure 6. Some differences can be seen between the spectra of the alpha- and beta-irradiated samples. First, for the samples measured without any additional heat treatment (marked on the graphs as 23 °C), the ratio of F_3_^+^ to F_2_ emission is higher for the alpha-irradiated sample (the ratio is 0.22) than for the sample irradiated with beta radiation (the ratio is 0.14). The reason may be similar to that deduced in the case of the absorption spectra: alpha particles deposit higher local doses, and a higher dose favors the creation of more complex centers. However, it is also possible that this difference is due to the fact that alpha particles deposit their energy only in a subsurface part of the sample. It has been reported that F_3_^+^ color centers are formed relatively more easily in the near-surface part of the crystal than in the bulk [31].

**Figure 7:**
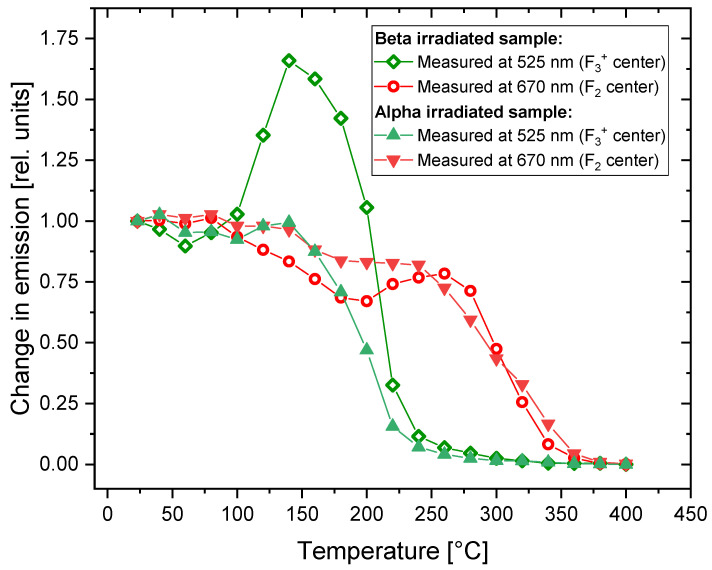
Relative changes in PL intensity at various temperatures with respect to the values at RT measured for two bands at 525 nm and 670 nm after the treatment according to Method 1. Changes were measured for the beta-irradiated (30 kGy) and alpha-irradiated (fluence 3.4 × 10^12^ cm^−2^) samples.

In Figure 7, the changes in photoluminescence emission after applying Method 1 to the irradiated samples are presented. The most noteworthy effect is the significant increase in the photoluminescence emission at 525 nm occurring at temperatures ranging from 100 °C to 200 °C for the beta-irradiated sample. The maximum enhancement of about 1.7 is reached for 140 °C. For the alpha-irradiated crystal, the effect is very weak, taking the shape of only a small local maximum. In this case, the PL intensity at 525 nm remains at almost the same level up to the temperature of 140 °C and then rapidly decreases. After being heated at 240 °C, practically no emission at that wavelength is present in the both alpha- and beta-irradiated samples. The decay of photoluminescence associated with F_3_^+^ centers was observed by Voitovich et al. [19] for similar temperatures.

The emission related to the F_2_ centers decreases up to the temperature of 200 °C and this decrease is steeper for the beta-irradiated crystal. At higher temperatures, we observe an increase in PL emission at 670 nm for the beta-irradiated sample, leading to a local maximum at 270 °C. For the alpha-irradiated sample, there is a kind of plateau in a similar temperature range. Above the temperature of 350 °C, the PL intensity reaches zero for both samples.

By comparing Figure 7 and Figure 4, we can see that the temperature at which the F_2_ and F_3_^+^ centers disappear does not depend on the type of radiation and in both cases is much lower for the F_3_^+^ centers (240 °C) than for the F_2_ centers (350 °C). However, a completely different behavior can be observed for the F centers (see Figure 4): they disappear after crystals are heated to lower temperatures for the beta-irradiated samples (300 °C) compared to the alpha-irradiated samples (380 °C).

**Figure 8:**
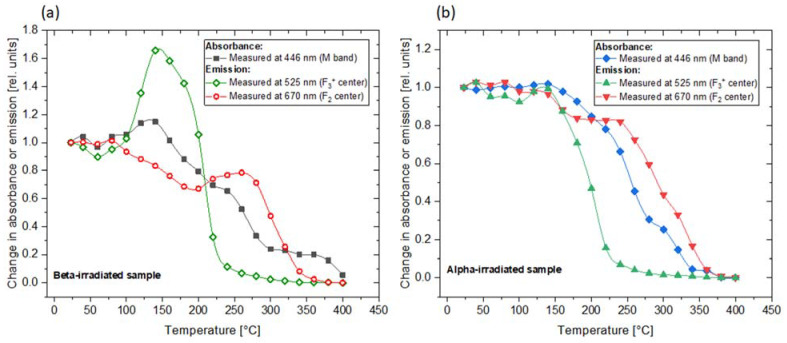
Comparison of the changes in absorption and PL intensity with respect to the values at room temperature measured for: (**a**) sample irradiated with beta radiation (30 kGy) and (**b**) sample irradiated with alpha particles (fluence 3.4 × 10^12^ cm^−2^) and subjected to Method 1.

A comparison of the changes observed in the absorption and photoluminescence emission spectra is presented in Figure 8. It is worth noting that the temperature of the heat treatment for which we observe the increase in PL emission at 525 nm corresponds to the maximum absorbance measured for the M band. This indicates that the enhancement of the 525 nm emission band is caused by the creation of new F_3_^+^ centers. In the case of the 670 nm emission, there is no distinct maximum in the absorption spectrum, which corresponds to the local maximum of PL. However, a small irregularity (a less-steep decrease in absorbance) may be noticed at temperatures of 250–270 °C.

**Figure 9:**
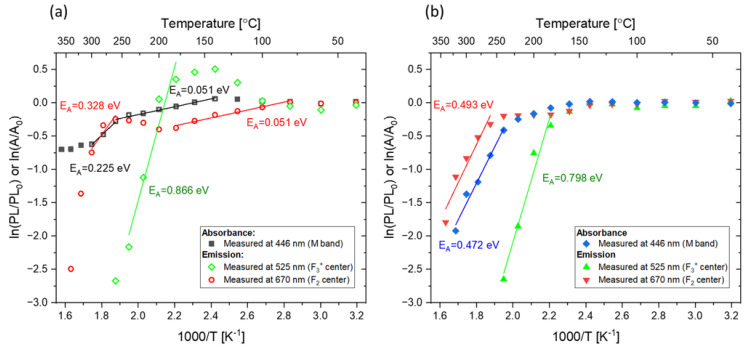
Arrhenius analysis of the changes in absorption and PL intensity with respect to the values at room temperature measured for: (**a**) sample irradiated with beta radiation (30 kGy) and (**b**) sample irradiated with alpha particles (fluence 3.4 × 10^12^ cm^−2^) and subjected to Method 1.

An Arrhenius analysis was performed for the data presented in Figure 8. The relationships $\mathrm{PL}={\mathrm{PL}}_{0}\exp\left(-\frac{E_{A}}{\mathrm{kT}}\right)$ and $A=A_{0}\exp\left(-\frac{E_{A}}{\mathrm{kT}}\right)$ were assumed, where ${\mathrm{PL}}_{0}\;$ and $A_{0}$ are PL intensity and absorbance, respectively, before heat treatment; $E_{A}$ is the activation energy; and T is the temperature in Kelvins. In the graphs presented in Figure 9, the slopes that can be expressed by a single exponent have been fitted to the experimental data and the activation energies have been calculated. It can be seen that while for the alpha-irradiated sample we observe a steady decrease in both absorbance and PL intensity, for the beta-irradiated sample, a more complex behavior is present. In both cases, however, the activation energies related to the decrease in F_2_ PL intensity and M-band absorbance for the heating procedure at temperatures exceeding 250 °C are similar. This can be explained by the lack of F_3_^+^ centers, which disappear at much lower temperatures than F_2_ centers. For that reason, after the heat treatment at high temperatures, the absorbance of the M band is exclusively connected to the F_2_ centers.

For the beta-irradiated sample, the absorbance data measured at 446 nm for temperatures above 300 °C were excluded from the analysis. After the heat treatment at these temperatures, there is no distinct peak at 446 nm, but a broad tail extending over a wide range of wavelengths is visible instead (which can be seen in Figure 2, inset). It is most probably not related to the discussed color centers but to other defects instead, e.g., Li colloids [22].

#### 3.1.3. Fluorescent Tracks

**Figure 10:**
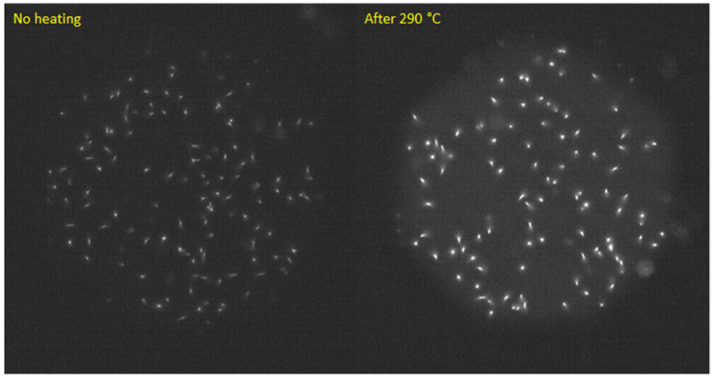
Microscopic fluorescent images registered with the same LiF crystal (acquisition time 2 s) before and after heat treatment at 290 °C. The sample was irradiated with Am-241 source (particle fluence around 1.60 × 10^6^ cm^−2^). The increase in the track intensity is about 2.5 times.

As mentioned in Section 1, the main motivation for which the present study was undertaken was the observed increase in the intensity of the fluorescent nuclear tracks after heating the LiF crystals [26]. This effect occurs after the treatments at temperatures between 200 °C and 300 °C and reaches the maximum of a 2.5-fold increase in track intensity at 290 °C. In Figure 10, the microscopic images of an alpha-irradiated sample before and after heat treatment at 290 °C are presented. The tracks in Figure 10 were registered with a long-pass filter ET570lp (red emission). We were not able to observe any tracks before or after heat treatment while using a bandpass filter HQ 535/30 (green emission). This means that for small doses of alpha radiation, there is no increase in the signal in the green part of the PL spectrum or this increase is not large enough to make tracks visible even while using long acquisition times.

**Figure 11:**
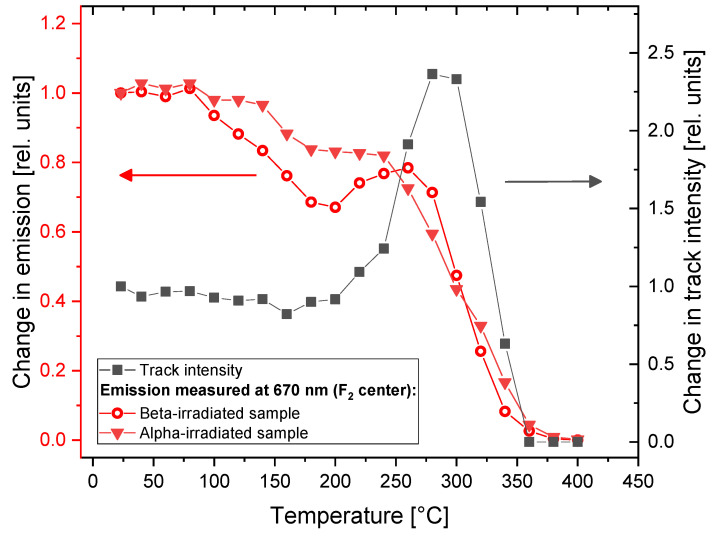
Relative changes in PL intensity for the beta-irradiated sample (30 kGy) and the sample irradiated with alpha particles, fluence 3.4 × 10^12^ cm^−2^ (left axis), in comparison to changes in fluorescent tracks intensity observed for the sample irradiated with a low fluence (1.6 × 10^6^ cm^−2^) of alpha particles (right axis). Arrows and plot colors indicate the applicable axes.

Figure 11 compares the changes observed in the photoluminescence emission spectra of the samples submitted to Method 1 with the results of our previous studies on fluorescent track intensity [26]. The measurements of the samples’ spectra confirm our previous observations that the very prominent effect of the enhancement of photoluminescence emission (measured as track intensity for the samples irradiated with low fluences of alpha particles) is not present for the LiF crystals irradiated with high doses of radiation (alpha fluence above 10^9^ cm^−2^). At the temperature of heat treatment of 290 °C, for which we observe the maximum increase in track intensity, the PL emission of the samples irradiated with high doses of beta and alpha radiation is at the level of 0.5 of their initial value (without any heat treatment). Although there is a small local maximum of emission for a beta-irradiated sample, its location does not agree perfectly with the maximum of track intensity (it is present for lower temperatures of heat treatment).

### 3.2. Method 2

#### 3.2.1. PL Emission Spectra

**Figure 12:**
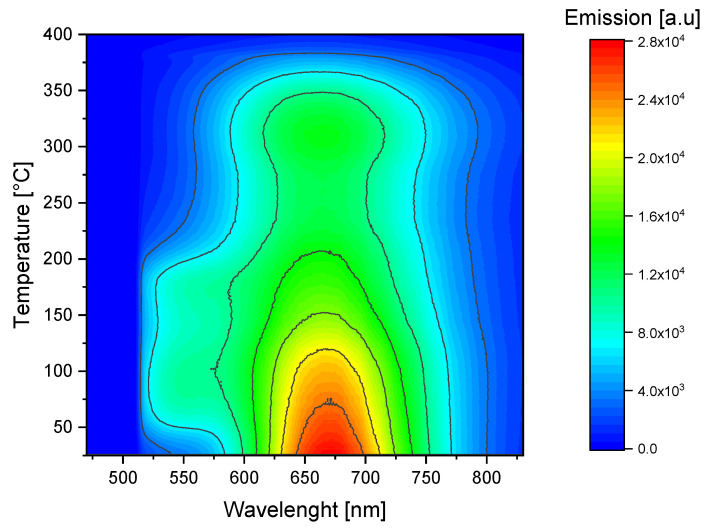
Photoluminescence emission spectra measured while heating the LiF crystal (sample submitted to Method 2). Excitation at 440 nm. The sample was irradiated with 8 kGy of beta radiation from Sr-90/Y-90 source. The spectrum is clipped from the low wavelength side by the applied filter.

**Figure 13:**
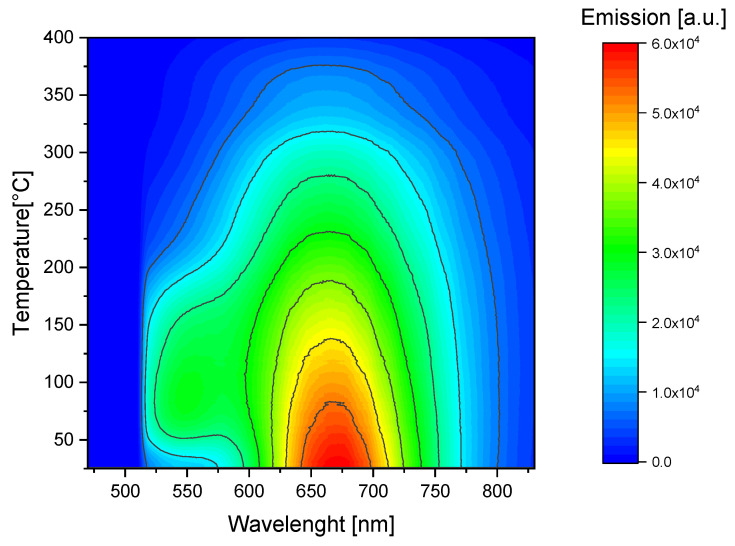
Photoluminescence emission spectra measured while heating the LiF crystal (sample submitted to Method 2). Excitation at 440 nm. The sample was irradiated with Am-241 alpha particles, fluence 10^12^ cm^−2^. The spectrum is clipped from the low wavelength side by the applied filter.

Figure 12 and Figure 13 show the changes in the photoluminescence emission spectra that were registered while the sample was heated (Method 2). Measurements were conducted for the sample irradiated with 8 kGy of beta radiation from the Sr-90/Y-90 source (Figure 12) and for the sample irradiated with alpha particles from the Am-241 source, with a fluence of 10^12^ cm^−2^ (Figure 13).

For both types of radiation, the highest signal was detected for temperatures close to room temperature (up to about 70 °C) for wavelengths within the range from 650 nm to 700 nm, i.e., the emission band related to F_2_ color centers. We can, therefore, conclude that carrying out measurements of the red photoluminescence emission at elevated temperatures offers no advantages from a practical point of view.

**Figure 14:**
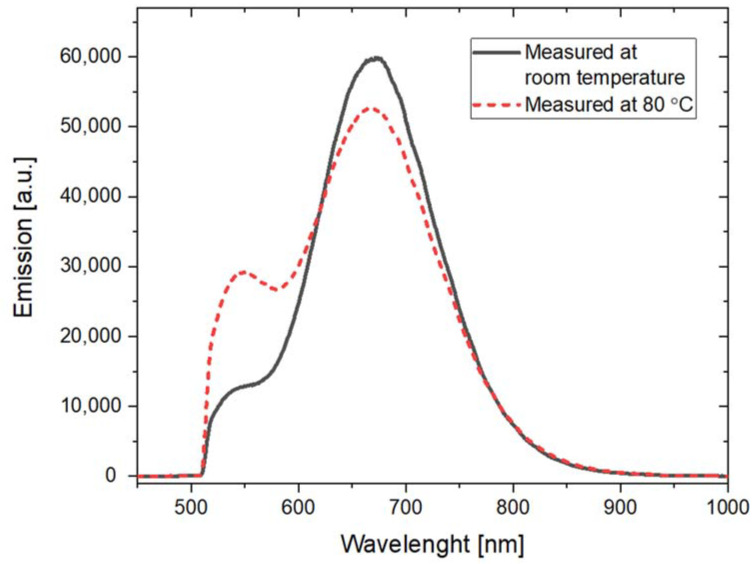
Photoluminescence emission spectra measured at room temperature and 80 °C for the sample irradiated with the alpha particles from Am-241 source (fluence 10^12^ cm^−2^). Excitation at 440 nm.

A different situation can be observed for the case of the emission peak at 525 nm, which is related to F_3_^+^ color centers. For this wavelength range, there is a clear increase in PL emission for temperatures from around 50 °C to 200 °C in comparison to the room temperature (RT) measurements. The PL spectra measured for the alpha-irradiated sample at room temperature and at 80 °C are compared in Figure 14. By comparing these two spectra, we can clearly see the increase in photoluminescence measured for the wavelength corresponding with the F_3_^+^ color centers (around 525 nm). A small decrease in the photoluminescence measured for a peak with a maximum at 670 nm is also present. Similar behavior was also observed for the beta-irradiated sample.

**Figure 15:**
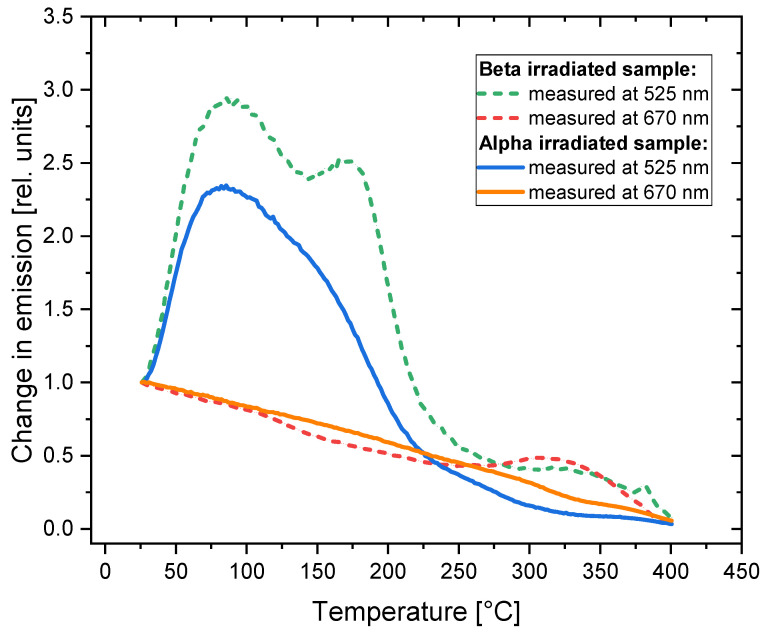
Changes in PL intensity with respect to the values at room temperature measured while heating the sample (the sample submitted to Method 2). Changes were measured for the sample irradiated with 8 kGy of beta radiation from the Sr-90/Y-90 source and for the sample irradiated with alpha particles from the Am-241 source (fluence 10^12^ cm^−2^). All data are normalized to the respective values measured at RT.

The relative changes in the photoluminescence emission measured at 525 nm and 670 nm are presented in Figure 15. Notably, although the relative changes for a signal measured at 525 nm are larger than for 670 nm, the absolute value of a photoluminescence signal is much higher for a wavelength of 670 nm.

For both types of radiation, the PL signal measured at 525 nm reaches the highest value at a temperature of around 80 °C. It is worth noting that this increase is much higher for Method 2 than the similar increase for Method 1 (see Figure 7), corresponding to a factor of 3 compared to a factor of 1.7 for the beta-irradiated samples. For the beta-irradiated sample, two distinct maxima are present (the second one at around 180 °C). For higher temperatures, the signal disappears very abruptly. The signal increase for the alpha-irradiated sample is smaller and exhibits only one evident peak at 80 °C. The differences in the signal increase for the samples irradiated with beta and alpha radiation may be caused by the different radiation doses or by the superficial nature of the alpha particles’ interaction with the crystal (penetration depth below 10 µm), which is similar to what was discussed for Method 1.

The second peak, present at 180 °C for the beta-irradiated sample, likely corresponds to the maximum that was observed at a temperature of 140 °C for the beta-irradiated sample subjected to Method 1 (Figure 7 and Figure 8). A small shift to a higher temperature for Method 2 may be explained by the different heating conditions; while for Method 1 a sample spends 3 min at the set temperature, for Method 2, the measurements are conducted only during heating, without maintaining any temperature. Therefore, it may be assumed that the origin of this peak is due to the creation of new F_3_^+^ centers.

The first peak at 80 °C seems to be related to a different mechanism than that at 180 °C. This peak is absent for Method 1, which indicates that it occurs only when an LiF crystal remains at elevated temperature during PL measurements. Therefore, it is very unlikely that the observed effect is related to the creation of new F_3_^+^ color centers. It seems that this effect is most probably caused by the mitigation of the process competitive to the radiative emission associated with F_3_^+^ color centers. The existence of a nonradiative process in the optical cycle of the F_3_^+^ color center in LiF has been reported, and it is connected to the metastable triplet state [32]. The F_3_^+^ color center can be excited from the ground state to the first excited state. After the relaxation time has elapsed, it may return to the ground state with PL emission. However, a sizable fraction of the excited centers may decay via a nonradiative transition to the ground triplet state and then by another nonradiative transition to the ground singlet state. This process decreases the efficiency of radiative emission. Temperature affects the probability of transition into a triplet state as well as the lifetime of the triplet state [33]. Based on the research published so far, it seems that the most likely explanation for the effect we observed is the reduction in the lifetime of the triplet state, which increases the concentration of the singlet-state F_3_^+^ color centers.

In the case of the 670 nm emission, no increase in the signal intensity was observed. There is a small local maximum at 320 °C for the beta-irradiated sample, which may correspond to the local maximum present for Method 1 at 260 °C (Figure 7). The shift to higher temperatures may again be caused by the different heating conditions. No such local increase in the 670 nm emission was observed for the samples irradiated with alpha particles, whether subjected to Method 1 or Method 2.

**Figure 16:**
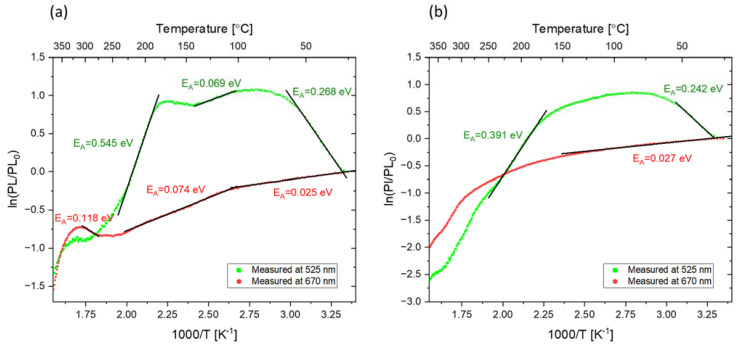
Arrhenius analysis of the changes in PL intensity with respect to the values at room temperature measured while heating the sample (the sample submitted to Method 2). Measured for: (**a**) the sample irradiated with 8 kGy of beta radiation from the Sr-90/Y-90 source and (**b**) the sample irradiated with alpha particles from the Am-241 source (fluence 10^12^ cm^−2^). All data are normalized to the respective values measured at RT.

For Method 2, like Method 1, an Arrhenius analysis was performed. In Figure 16, fitted slopes and calculated activation energies are presented. The activation energies, corresponding to an increase in PL intensity in the green part of the spectra (F_3_^+^ centers) at low temperatures, are almost the same for the beta- and alpha-irradiated samples. This suggests that the process responsible for the enhancement of the signal does not depend on the type of radiation or the dose used. This seems to confirm our hypothesis that changes in PL intensity at these temperatures are caused by different mechanisms than the enhancement observed for PL intensity connected to F_3_^+^ color centers observed after applying Method 1. In addition, for temperatures lower than 120 °C, the same activation energy was calculated for the decrease in PL intensity in the red part of the spectrum (related to F_2_ color centers) for the alpha- and beta-irradiated samples. At temperatures higher than 150 °C, noticeable differences were observed between the graphs and activation energies for the samples irradiated with different types of radiation. They are most probably caused by the effects that were observed after applying Method 1.

#### 3.2.2. Fluorescent Tracks

The described increase in the green part of the photoluminescence spectrum during heating is a desirable effect; as for microscopic observation, we are normally not able to observe any tracks at that spectral range. The concentration of F_3_^+^ color centers is usually very low and, even for a very high acquisition time, the photoluminescence signal cannot be distinguished from the background.

**Figure 17:**
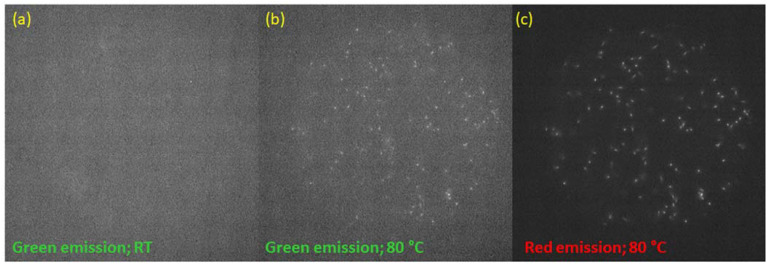
Microscopic images registered for the sample irradiated with alpha particles (Am-241 fluence 1.6 × 10^6^ cm^−2^): (**a**) read-out at room temperature with a bandpass filter HQ 535/30 (green emission), with an acquisition time of 10 s; (**b**) read-out at 80 °C with a bandpass filter HQ 535/30 (green emission), with an acquisition time of 10 s; (**c**) read-out at 80 °C with a long-pass filter ET570lp (red emission), with an acquisition time of 2 s.

To determine whether the effect of the increase in the PL signal in the green part of the spectrum also occurs for small doses of radiation, we performed observations with a fluorescent microscope using a bandpass filter HQ 535/30 at a temperature of 80 °C. Example images are shown in Figure 17b.

It can be seen that the read-out at elevated temperatures indeed enables track observations in the green part of the spectrum as well. Therefore, this is a different situation than in the case of Method 1, where the significant increase in PL emission at the 525 nm band still had not enabled the visualization of the tracks in this part of the spectrum. It is not possible to quantitatively determine the degree of the relative increase in the photoluminescence signal in this spectral range, as tracks of alpha particles are completely invisible for observations at room temperature in this spectral range.

## 4. Conclusions

In this work, we studied the influence of thermal treatment at temperatures ranging from RT to 400 °C on absorption and PL emission spectra as well as fluorescent nuclear tracks in the irradiated LiF crystals. The performed investigations allowed us to identify the following significant effects induced by the thermal treatment on the photoluminescence of LiF:

- Heating at temperatures ranging between 100–200 °C increases the concentration of F_3_^+^ centers. The effect is visible both in the absorption and emission spectra and is much more significant for beta-irradiated than alpha-irradiated crystals. However, the increase in PL emission is too small to enable microscopic observation of fluorescent tracks in the green part of the spectrum.
- F_3_^+^ PL emission is very significantly increased when a measurement is performed at temperatures around 80 °C (factor 3), which is presumably due to the lower probability of the competitive, nonradiative process connected with the existence of the triplet state, which is present in the optical cycle of this color center. Such elevated temperatures of measurement enable the observation of fluorescent nuclear tracks in the F_3_^+^ green part of the spectrum. In the case of the main F_2_ red emission, raising the temperature of PL measurements does not lead to any increase in the signal.
- Heating at around 290 °C substantially increases F_2_ PL in the case of fluorescent track measurements (factor 2.5). The supposed cause is the creation of new F_2_ centers. However, this effect is barely visible in the emission spectrum (a small local maximum is present at a slightly lower temperature) and unnoticeable in the absorption spectrum. These results are not surprising, as previous investigation of fluorescent tracks showed that the strength of the effect decreases with the increasing dose, and spectral measurements required irradiation with much higher doses than the track observations. The mechanism behind these effects remains to be revealed and further investigations in this direction are planned.

## Data Availability

Not applicable.
